# Copy Number of Human Ribosomal Genes With Aging: Unchanged Mean, but Narrowed Range and Decreased Variance in Elderly Group

**DOI:** 10.3389/fgene.2018.00306

**Published:** 2018-08-07

**Authors:** Elena M. Malinovskaya, Elizaveta S. Ershova, Vera E. Golimbet, Lev N. Porokhovnik, Nataliya A. Lyapunova, Serguey I. Kutsev, Natalia N. Veiko, Svetlana V. Kostyuk

**Affiliations:** ^1^Research Centre for Medical Genetics (RCMG), Moscow, Russia; ^2^Mental Health Research Center, Moscow, Russia

**Keywords:** ribosomal DNA, rDNA copy number, ribosomal RNA, methylated rDNA, ribosomal genes

## Abstract

**Introduction:** The multi-copied genes coding for the human 18, 5.8, and 28S ribosomal RNA (rRNA) are located in five pairs of acrocentric chromosomes forming so-called rDNA. Human genome contains unmethylated, slightly methylated, and hypermethylated copies of rDNA. The major research question: What is the rDNA copy number (rDNA CN) and the content of hypermethylated rDNA as a function of age?

**Materials and Methods:** We determined the rDNA CN in the blood leukocyte genomes of 651 subjects aged 17 to 91 years. The subjects were divided into two subgroups: “elderly” group (E-group, *N* = 126) – individuals over 72 years of age (the age of the population’s mean lifetime for Russia) and “non-elderly” group (NE-group, *N* = 525). The hypermethylated rDNA content was determined in the 40 DNA samples from the each group. The change in rDNA during replicative cell senescence was studied for the cultured skin fibroblast lines of five subjects from NE-group. Non-radioactive quantitative dot- and blot-hybridization techniques (NQH) were applied.

**Results:** In the subjects from the E-group the mean rDNA CN was the same, but the range of variation was narrower compared to the NE-group: a range of 272 to 541 copies in E-group vs. 200 to 711 copies in NE-group. Unlike NE-group, the E-group genomes contained almost no hypermethylated rDNA copies. A case study of cultured skin fibroblasts from five subjects has shown that during the replicative senescence the genome lost hypermethylated rDNA copies only.

**Conclusion:** In the elderly group, the mean rDNA CN is the same, but the range of variation is narrower compared with the younger subjects. During replicative senescence, the human fibroblast genome loses hypermethylated copies of rDNA. Two hypotheses were put forward: (1) individuals with either very low or very high rDNA content in their genomes do not survive till the age of the population’s mean lifetime; and/or (2) during the aging, the human genome eliminates hypermethylated copies of rDNA.

## Introduction

Protein synthesis is the central event in the eukaryotic cell. Molecular machines termed ribosomes conduct the protein biosynthesis (translation). The human ribosome consists of two major components: ribosomal RNA (rRNA) of four types (18, 5.8, 28, and 5S) and approximately 70–80 ribosomal proteins. The rRNA and ribosomal proteins form the structure consisting of two subunits, which performs the process of translation (Khatter et al., 2015).

**FIGURE 1:**
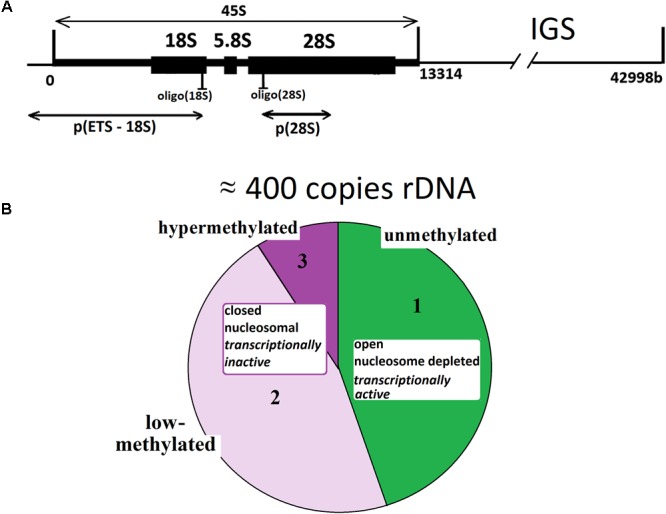
**(A)** A scheme of the human ribosomal repeat. Segments of 18S rDNA and 28S rDNA analyzed with NQH are shown. **(B)** rDNA fractions within the human ribosomal gene arrays. Fraction 1 is transcriptionally active rDNA copies. Fraction 2 is inactive, low-methylated rDNA copies. Fraction 3 is hypermethylated rDNA copies.

Multiple copies present genes for rRNA (rDNA) in human genome. The diploid human genome contains ∼400 copies of a 43-kb rDNA unit tandemly arrayed in nucleolar organizer regions (NORs) on the five acrocentric chromosomes. Each unit contains 13.3 kb of a sequence encoding the 28, 5.8, and 18S rRNAs (45S rRNA) and a non-coding intergenic spacer (IGS) (McStay and Grummt, 2008), **Figure 1A**. Together with the 5S rRNA (encoded by genes located on chromosome 1), these rRNAs form the nucleic acid backbone of the ribosome.

The set of data shows that rDNA chromatin can exist in at least three distinct states (Santoro and Grummt, 2001; Santoro et al., 2002; Grummt and Pikaard, 2003; Stefanovsky and Moss, 2006; Grummt, 2007; Gagnon-Kugler et al., 2009; Felle et al., 2010; Lyapunova and Veiko, 2010; Xie et al., 2012; Hamperl et al., 2013; Diesch et al., 2014; Kusnadi et al., 2015; Hung et al., 2017). The fraction of states 1 (**Figure 1B**) is transcriptionally active rDNA copies. The transcriptionally active rDNA is euchromatic, hypomethylated at CpG sites, and marked with histone modifications generally associated with transcriptionally active nucleoplasmic genes (i.e., H3K4me3 and H3K9ac) (Hamperl et al., 2013). The copy number of transcriptionally active rDNA copies is roughly estimated as 30% to 50% of the total rDNA copies in the genome (Conconi et al., 1989; French et al., 2003; Lyapunova and Veiko, 2010; Lyapunova et al., 2013). Inactive non-methylated or poorly methylated rDNA copies form fraction 2 are believed to be found in the same structures of the nucleolus as is transcriptionally active copies and necessary for normal operation of the nucleolus. The copies within fraction 2 are inactive, apparently, due to methylation of some CpG sites in the rDNA promotor region (Santoro and Grummt, 2001; Grummt and Pikaard, 2003; Grummt, 2007; McStay and Grummt, 2008; D’Aquila et al., 2017). These copies contain nucleosomes (close conformation). Fraction 3 consists of inactive hypermethylated rDNA copies. These copies are located on the periphery of the nucleolus and constitute the heterochromatin that encompasses the nucleolus. The hypermethylated rDNA copies can be found in the genomes of about 20% of individuals (Lyapunova and Veiko, 2010; Lyapunova et al., 2013).

There are number of association studies between rDNA content in genome and aging, and the results have been conflicting. The rDNA gene copy number (rDNA CN) was found to be associated with aging. Johnson and Strehler (1972) proposed a hypothesis that aging-related loss of rDNA in post-mitotic cells, including muscles and neurons, leads to aging associated dysfunction simply by insufficient ribosome supply and translational failure. Age-dependent loss of rDNA has been demonstrated in beagle dog brain tissues, in human myocardium and cerebral cortex (Strehler and Chang, 1979; Strehler et al., 1979) and in mouse brain spleen and kidney tissues (Gaubatz and Cutler, 1978). Subsequent experiments demonstrated an age-dependent loss of rDNA in polyclonally stimulated peripheral lymphocytes (Das et al., 1986). Work that is more recent suggested an age-dependent decrease in rDNA content in human adipose tissue that was limited to 5.8 and 28S regions (Zafiropoulos et al., 2005).

However, some studies did not confirm observations of aging-associated changes in the genomic rDNA content. The number of rRNA genes of mouse heart was not found to change significantly as a function of age (Peterson et al., 1984). The copy number of the rRNA genes did not change during serial passage of the rat embryo fibroblasts, the human skin fibroblasts, or the WI-38 cells (Peterson et al., 1984; Halle et al., 1997; Machwe et al., 2000). Probing rRNA-coding regions of rDNA in the human cerebral cortex revealed no effects of normal aging on the rDNA content (Hallgren et al., 2014).

The meta-analysis of reports cited above has shown that the result of determination of the rDNA abundance in young and old organisms or cell lines largely depends on the research technique. While a technique based on hybridization with DNA probes often suggests no decrease in rDNA copy number (rDNA CN) during natural and replicative senescence, qPCR and other, older approaches lead to the opposite conclusion. In addition, small numbers of samples were tested in the cited studies.

Thus, the first task of our study was an assay of variability of rDNA *CN* in the genomes of large enough groups of subjects of various age (totally, 651 subjects). Within the framework of this task, we had to choose the optimum method for rDNA quantification in a large number of DNA samples. Our most recent studies have shown that the method of non-radioactive quantitative hybridization (NQH) yields more accurate and reproducible results for rDNA content, than qPCR. The difference between the two techniques are especially prominent when assaying damaged DNA samples [DNA derived from old cells, from the patients with high level of oxidative stress, oxidized DNA (Chestkov et al., 2018), and cell-free DNA (Korzeneva et al., 2016)].

In the field of aging epigenetics, there are few publications, which report the studies of changes in rDNA methylation pattern in human aging. An age-related increase was found in rDNA methylation in tissues of differently aged mice and in sperm and liver of male rats (Swisshelm et al., 1990; Oakes et al., 2003). The *in vitro* senescence of human fibroblasts is accompanied by an increase in cytosine methylation within rDNA genes (Machwe et al., 2000).

However, the analysis performed by other authors showed that the methylation state of the rRNA genes did not change significantly with increasing cumulative population doublings of the rat embryo fibroblasts (Halle et al., 1997). The authors of the study as of year 2017 applied a bisulfite-based approach that relies on base-specific cleavage and mass spectrometry to measure the methylation frequencies of CpG dinucleotides located within different *cis*-elements (spanning from -189 to +47 bp) in the promoter of human rRNA genes. No consistent statistically significant association was found between the methylation level of the analyzed CpG-sites and the subject’s age (D’Aquila et al., 2017). However, one may suppose that two oppositely directed processes occur during aging: augmentation of the methylation of rDNA copies that constitute fraction 2 (low-methylated, inactive) and loss of hypermethylated rDNA copies belonging to fraction 3 (**Figure 1B**). As a result, the total detected methylation level of the promotor region remains constant. There is, however, no report of a special research of changes in the hypermethylated rDNA content (fraction 3, **Figure 1B**) during the process of human aging.

Thus, the second task of our study was determination of the content of hypermethylated rDNA copies in leukocyte DNA isolated from subjects of different age groups (natural aging) and in cultured skin fibroblast DNA at early and late cultivation stages (replicative senescence).

## Materials and Methods

### Population Samples

The dataset included 651 unrelated individuals inhabiting Moscow and the Moscow region (545 men/106 women) with a median age of 48 years (17–91 years). The participants were tentatively divided into two groups. The non-elderly (NE) group included 525 subjects aged 17 to 71 years (a median age of 38 years), and the elderly (E) group included 126 subjects aged 72 to 91 years (a median age of 83 years). The personnel of Moscow nursing home for war veterans conducted collecting peripheral blood samples from most old subjects (80 to 91 years old). Collecting peripheral blood samples from the other subjects aged 17–79 was conducted by the personnel of Research Centre for Medical Genetics and Mental Health Research Center (Moscow). The investigation was carried out in accordance with the latest version of the Declaration of Helsinki and approved by the Regional Ethics Committee of RCMG (Approval # 5). All participants signed an informed written consent to participate after the nature of the procedures had been completely explained to them. Among the subjects, there were no patients with genetic diseases, which certainly shorten the lifetime.

### DNA Samples

Five milliliter of blood was collected from a peripheral vein of the subject using a syringe flushed with heparin (0.1 mL/5 mL blood) under strict aseptic conditions. The DNA isolation method has been described in detail previously (Veiko et al., 2003). Briefly, DNA was isolated from 5 mL of blood. Five milliliter of the solution containing 2% sodium lauryl sarcosylate, 0.04 M EDTA, and 150 μg/mL RNAse A (Sigma, United States) were added to the fresh blood for 45 min (37°C), then were treated with proteinase K (200 μg /mL, Promega, United States) for 24 h at 37°C. The lysate samples were extracted with an equal volume of phenol, phenol/chloroform/isoamyl alcohol (25:24:1), and chloroform/isoamyl alcohol (24:1), respectively. DNA was precipitated by adding 1/10 volume of 3 M sodium acetate (pH 5.2) and 2.5 volume of ice-cold ethanol. For the extraction procedure, only freshly distilled solvents were used. Phenol was stabilized with 8-hydroxyquinoline. Finally, the DNA was collected by centrifugation at 10,000 *g* for 15 min at 4°C, washed with 70% ethanol (v/v), dried, and dissolved in water. The DNA concentration and purity were determined spectrophotometrically. The final DNA quantification was performed using PicoGreen dsDNA quantification reagent from Molecular Probes (Invitrogen, Carlsbad, CA, United States). The assay indicated a linear correlation between dsDNA quantity and fluorescence within a wide range. The DNA concentration in the samples was calculated according to a DNA standard curve. We used EnSpire equipment (Finland) with excitation and emission wavelengths of 488 and 528 nm, respectively.

### Non-radioactive Quantitative Hybridization

#### The DNA Concentration

The success of NQH depends on the accurate quantification of the DNA content. We perform DNA quantification in two different steps. The first one gives a rough estimate of the initial amount of DNA in each sample by the method of UV spectroscopy. At the end of the first step, the amount of DNA needed to make a 50 ng/μL solution of DNA is calculated. The final DNA quantification is performed fluorimetrically using the PicoGreen dsDNA quantification reagent by Molecular Probes (Invitrogen, Carlsbad, CA, United States). The assay displays a linear correlation between dsDNA quantity and fluorescence within a wide range of concentrations. The DNA concentration in the sample is calculated according to a DNA standard curve. We use EnSpire equipment (Finland) at excitation and emission wavelengths of 488 and 528 nm, respectively.

#### The Oligo-Probes

For the detection of human rDNA (GenBank Accession No. U13369, Gonzalez and Sylvester, 1995), a mixture of rDNA probes was used (**Figure 1A**): oligo(18S) biotine-CTGTAATGATCCTTCCGCAGGTTCACCTAC and oligo(28S) biotine-TATCGGTCTCGTGCCGGTATTTAGCCTTAG.

#### The DNA-Probes

DNA probes used in our research are shown in **Figure 1A**. The p(ETS-18S)–EcoR1 fragment of rDNA 5.8-kb long (from -515 till 5321 relative to the transcription initiation point) was cloned in pBR322 plasmid. The p(28S)–Rsa1 fragment of 28S rDNA 2413 bases long (from 8289 till 10702) was cloned in pBR322 plasmid. The DNA-probe was biotinylated using the nick translation kit (Biotin NT Labeling Kit, Jena Bioscience GmbH). For the detection of rDNA CN, a mixture of the two probes was used.

Of 651 DNA samples totally studied, 530 samples were analyzed using biotinylated probes p(ETS-18S) and p(28S). For the other samples, biotinylated probes oligo(18S) and oligo(28S) were applied. Special experiments have shown that the results of quantification of rDNA copies do not depend on the probe type. However, when longer probes are used, the hybridization signal intensities are higher and less amounts of DNA are required for the hybridization.

#### The Method of Quantitative Non-radioactive Hybridization

It was specified in details previously in three publications (Veiko et al., 2003; Korzeneva et al., 2016; Chestkov et al., 2018). Briefly, the denatured DNA samples (from 4 to 6 dots per each sample) were applied to a filter (Optitran BA-S85, GE healthcare). Standard samples of the genomic DNA (50 ng/mL) with a known content of the rDNA were applied to the same filter, in order to plot a calibration curve for the dependence of the signal intensity on the number of rDNA copies in a particular sample. For calibration, we use six samples of human DNA with a known number of copies of rDNA. This amount was previously determined by direct comparison of the content of the rDNA fragment in a sample of genomic DNA and in a model sample that contains a known number of molecules of the analyzed rDNA fragment (Southern blot hybridization method). Lambda phage DNA (50 ng/mL) was also applied to the same filter in order to control the non-specific signal. The filter was heated at 80°C in vacuum for 1.5 h. After hybridization was completed, the membrane filter was treated with a conjugate of streptavidin with alkaline phosphatase (Sigma) and was placed into a solution of substrates for alkaline phosphatase (NBT and BCIP). Upon the completion of reaction, the filter was washed with water and dried in the darkness. The dried filter was scanned. In order to assay the hybridization outcome, special software was used (“Imager 6,” RCMG, Moscow). The software determined the dot location, measured the nearest background signal, and calculated the integral dot intensity. Signals from several dots corresponding to the same sample were averaged. The mean and standard error were calculated. The rDNA content in a studied sample was calculated using the calibration curve equation. Relative standard error of the index CN(NQH) was 11 ± 8%.

### DNA Methylation Analysis

The level of rDNA methylation was estimated using methylation-sensitive restriction analysis (MSRA). One hundred nanograms of DNA were digested with Csp-6I (HpaII + Csp-6I) or (MspI + Csp-6I) in 20 μL of “Tango” buffer [1× Buffer composition: 33 mM Tris-acetate (pH 7.9 at 37°C), 10 mM magnesium acetate, 66 mM potassium acetate, 0.1 mg/ml BSA] according to the manufacturer’s instruction (18 h, 37°C). The DNA was precipitated by adding two volumes of ethanol in the presence of 3 M sodium acetate. The precipitate was then washed with 75% ethanol twice, then dried, and dissolved in water.

The qualitative assessment of methylation level (determination of methylation group) was performed with non-radioactive Southern blot analysis. The electrophoresis was conducted in 1.2% agarose gel. The transfer onto a nylon membrane was performed with vacuum-blot technique. Five microgram of DNA was put on each gel track. The quantitative assessment (determination of methylation index M) was conducted with NQH technique using p(ETS-18S) probe. Each value of the index *M* represents the average of three independent experiments.

### Cell Cultures

We used five long-term lines of diploid human dermal fibroblasts. Lines HSF–45, HSF–53, HSF–57, HSF–61, and HSF–66 were obtained from skin biopsies of healthy adult persons. The digits in each line denotation indicated the order number of the final passage, after which the cells stopped their division. The cells were cultured in Eagle’s medium supplemented with 10% fetal bovine serum at 37°C at saturation humidity in an atmosphere containing 3–5% CO_2_. The cells (about one-third of the total amount) were subcultured approximately once every 3 days, then cultured till subconfluent condition, and subcultured again. To isolate DNA from the cells, the technique described above for the blood was used. Quantification of rDNA and determination of methylation index M was performed using NQH technique.

### Statistical Analysis

All the findings reported here were reproduced at least two times as independent biological replicates. The descriptive statistics are listed in **Table 1**. The significance of the observed differences was analyzed using the non-parametric Mann–Whitney *U*-test or the Kolmogorov–Smirnov statistics. Linear regression analyses were carried out to evaluate the effect of rDNA CN as a function of age. Data were analyzed with StatPlus2007 Professional software^1^. All *p*-values were two-sided and considered statistically significant at *p* < 0.01.

**Table 1:** Abundance of rDNA CN in leukocyte DNA from subjects of different age.

Descriptive statistics	NE-group (17–71 years)	E-group (72–91 years)
*N*	525	126
Range	200–711^a^	272–541^a^
Mean ± SD	419 ± 110	396 ± 63
Median	410	394
Coefficient of variation	0.26^b^	0.16^b^

*^*a,b*^The differences are statistically significant (*p* < 0.001). NE, non-elderly; E, elderly; SD, standard deviation.*

## Results

### Validation of the Analytical Method Selected

At present, the main method for the quantification of various genome sequences is the generally acknowledge technique of qPCR. However, the rRNA-coding region of rDNA is a problematic object for the quantification using qPCR for several reasons, which are discussed in detail in our previous articles (Korzeneva et al., 2016; Chestkov et al., 2018). Briefly, the qPCR efficiency is affected by the tandem nature of rDNA repeats and the presence of self-complementary regions within the repeat unit, the heterogeneity of rDNA copies with regard to methylation. In addition, qPCR efficiency is affected by the high occurrence of Gn (*n* > 2) motifs, which have the lowest oxidation potential (von Sonntag, 2006), the existence of breaks in rDNA even in intact cells, and the reduced rDNA repair level. The situation worsens in case of quantification of rDNA in certainly damaged DNA samples. The dependence of rDNA CN, as determined with qPCR, on the DNA oxidation damage degree is non-linear. When the oxidation degree is low, rDNA abundance can be overestimated, while in case of high damage degree, it is substantially underestimated (Chestkov et al., 2018).

We found a substantial reduction of qPCR efficiency for rDNA as compared to standard gene B2M (1.85 vs. 1.99). Analysis of literature data has also shown that the efficiency of qPCR for rDNA is reduced. So the value *d* (Ct) = Ct_B2M_-Ct_rDNA_ varied from +5 down to -10 for rDNA even in young cells (Zafiropoulos et al., 2005). These data strongly suggest a very low qPCR efficiency for rDNA compared to the reference gene.

As we showed previously, quantification of damaged rDNA produced better results when the method of hybridization with oligonucleotide or long DNA probes was used in the non-radioactive variant (Korzeneva et al., 2016; Chestkov et al., 2018). Unlike the generally acknowledged qPCR, NQH technique is not applicable to unique (single-copy) genes. This method requires large amounts of DNA. However, for the NQH technique, the rDNA damage degree has no influence on the determined abundance of rDNA and is underestimated only in the samples with very high DNA damage degree (Chestkov et al., 2018).

**FIGURE 2:**
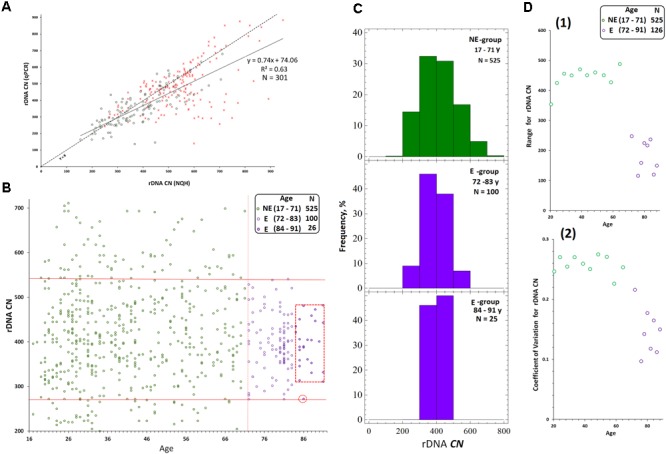
Determination of rDNA copy number in blood leukocyte DNA samples from 651 subjects. **(A)** Choice of the method of quantitative analysis for rDNA CN. Analysis of rDNA CN for healthy control (*N* = 122, green) and patients with schizophrenia (*N* = 179, red) with NQH (*X* axis) and qPCR (*Y* axis). The graph is based on the results of our previous study (Chestkov et al., 2018). **(B)** Association of rDNA CN (NQH) with age for NE-group (17 to 71 years old, *N* = 525), E-group (72 to 83 years old, *N* = 100), and E-group (84 to 91 years old, *N* = 26). **(C)** Distribution of rDNA copy number in the examined groups. **(D)** Association of the Range (1) and the Coefficient of variation (2) for rDNA CN with age for NE-group and E-group. The groups were divided into sub-groups according to the age. The age of the subjects in each NE-subgroup varied in the interval of 5 years and in E-subgroup – in the interval of 2 years. The *X* axis: average age values in each subgroup. The range and the coefficient of variation for NE-group are significantly greater than for E-group (*p* = 0.0004).

For instance, see **Figure 2A** that presents joint data obtained in our previous research on 301 DNA samples from blood leukocytes of healthy controls and schizophrenia cases (Chestkov et al., 2018). The vast majority of the control DNA samples and 80% of cases showed a good correlation between rDNA CN values obtained with the two methods. However, in 20% of samples isolated from the cases, the rDNA CN(NQH) values were significantly higher, than rDNA CN(qPCR). The decrease in qPCR efficiency is a consequence of oxidative DNA damage in schizophrenia patients. It is also known, that the efficiency of qPCR is reduced when quantifying cells from old individuals (Ploskonosova et al., 1999) and the DNA obtained from senescent cells (Chestkov et al., 2018).

That is why in this article we presented data obtained with NQH only, in spite of the fact that we usually apply both methods in our rDNA studies.

### Comparative Analysis of rDNA Abundance in Genomes of Human Subjects of Different Age

**Figure 2B** presents the dependence of rDNA CN on age for the population of Moscow and Moscow region residents (*N* = 651, 545 male/106 female subjects), and corresponding distributions for rDNA CN are shown in **Figure 2C**. The dependence of the range and the coefficient of variation for rDNA CN on age are shown in **Figure 2D**(1,2). **Table 1** contains the descriptive statistics. Mean rDNA CN values does not depend on age and sex and are virtually the same for each age group (*p* > 0.05). However, the subgroup of subjects aged 72 to 91 years (E-group, *N* = 126) contained no genomes harboring less than 272 or more than 541 rDNA CN. The range and the coefficient of variation were significantly lower in E-group (*p* < 0.001) compared to the subjects of younger age (NE-group, *N* = 525), **Figures 2B–D**(1,2) and **Table 1**. The range of variation of the rDNA CN narrows to the maximum extent for the people aged 83 to 91 years (median 396; range 311–482 rDNA CN for 25 out of 26 subjects, **Figures 2B,C**).

### Comparative Analysis of rDNA Methylation in the Genomes of E-Group and NE-Group

**FIGURE 3:**
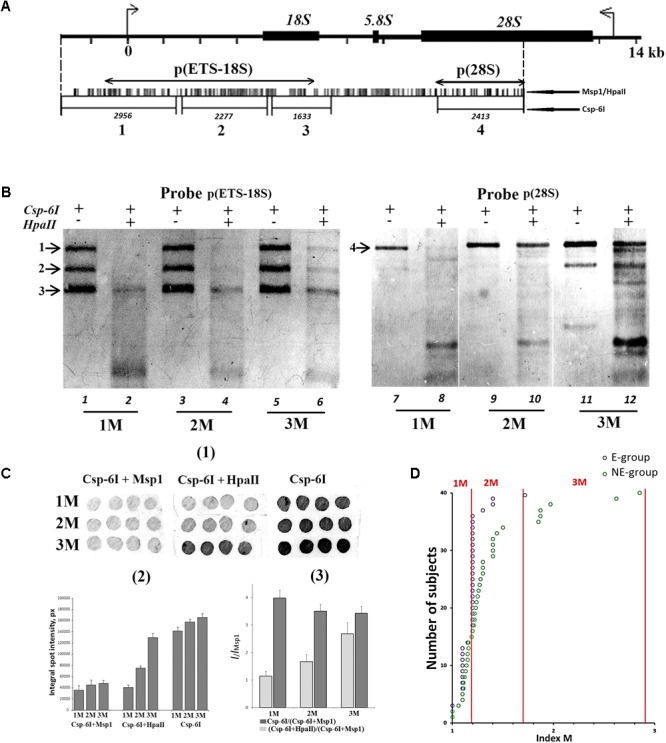
**(A)** A scheme of the human ribosomal repeat. Positions of sites for Msp1/HpaII and Csp-6I endonucleases are shown. The major rDNA fragments (1–4) are shown, which are formed due to cleavage by Csp-6I and detected with biotinylated probes p(ETS-18S) and p(28S). The fragment length is presented in base pairs (bp). **(B)** Examples of non-radioactive Southern blot analysis of three DNA samples with different quantities of hypermethylated rDNA copies in the genomes are demonstrated. Fragments 1–4 are shown. Samples that belong to 1M demonstrate no bands corresponding to fragments 1 and 2. In samples that belong to 3M, all the bands corresponding to fragments 1–4 can be clearly seen. **(C)** Examples of the use of NQH technique for the determination of rDNA methylation index (M). (1) An example of hybridization of DNA hydrolyzates containing various amounts of hypermethylated rDNA copies in the genome with the biotinylated probe p(ETS-18S). (2) Values of integral intensity of the hybridization signal (means for four dots and standard deviations are shown). (3) Ratio of integral intensities of signals (Csp-6I + HpaII) and (Csp-6I + Msp1) hydrolyzates (methylation index *M*). For comparison, ratio of integral intensities of signals (Csp-6I) and (Csp6I + Msp1) hydrolyzates is shown. **(D)** The number of DNA samples containing rDNA copies methylated by types 1M–3M in the samples belonging to E-group (*N* = 40, aged 80–91 years) and NE-group (*N* = 40, aged 17–60 years). Ranges of methylation index *M* for each methylation group are indicated in the figure. Each value of the index *M* represents the average of three independent experiments.

We randomly selected 40 DNA samples from NE-group (the chosen samples covered of range of 17–60 years old) and 40 DNA samples from E-group (covered a range of 80–91 years old). Quantification of hypermethylated copies in the genome (fraction 3, **Figure 1B**) was performed using NQH (**Figure 3**). The restriction endonucleases HpaII and MspI were used to study sequence-specific methylation patterns with Southern blot analysis. HpaII cleaves the sequence CCGG but is inhibited if the internal cytosine is methylated, whereas MspI cuts both CCGG and C^me^CGG efficiently. Simultaneously with HpaII or MspI, Csp-6I endonuclease was used (Rsa1, recognizes GTˆAC sites), which is not sensitive to cytosine methylation.

**Figure 3B** presents examples of Southern blot analysis. All the DNA samples can be arbitrarily divided into three groups. Group 1M had low frequency of methylation, especially in the promotor region (band 1, track 2, **Figure 3B**), and no rDNA copies hypermethylated within the whole length. Group 2M had small number of hypermethylated copies (about 10%). Group 3M had many hypermethylated copies (up to 30% of the total amount). Interestingly, copies with rearranged rDNA, in which changed positions of Csp-6I sites were detected (track 11, **Figure 3B**), often occurred in group 3M.

For the quantitative analysis of methylation index *M*, an approach was developed, which included dot-hybridization of (MspI + Csp-6I) and (HpaII + Csp-6I) hydrolysates with probes p(ETS-18S) or p(28S), see **Figure 3C**(1,2). The methylation index *M* was calculated as the ratio of hybridization signals of the (HpaII + Csp-6I) and (MspI + Csp-6I) hydrolysates [**Figure 3C**(3)]. For group 1M, the M index varied within a range of 1.02–1.2; for 2M, within a range of 1.3–1.7, and for 3M, within a range of 1.8–2.9. The analysis of 40 DNA samples from NE-group and 40 DNA samples from E-group has shown that in E-group, the frequency of carriers of 2M and 3M was lower than in NE-group (4 samples vs. 20 samples), **Figure 3D**. Thus, rDNA in the genomes of 90% of individuals older than 80 years contained practically no hypermethylated copies. Genomes of approximately 30% of individuals aged 17 to 60 years contained considerable amounts of hypermethylated rDNA copies.

### Changes of the Number of rDNA Copies During Replicative Senescence of Human Skin Fibroblasts

Five cultured lines of skin fibroblasts were derived from the donors selected from the NE-group. For the selection of skin donors, two criteria were applied: the number of rDNA copies in the genome and methylation index *M*, which was determined for the leukocyte DNA. **Table 2** presents the major parameters of the five fibroblast lines analyzed. Two of five fibroblast donors carried hypermethylated rDNA copies in the leukocyte DNA and were classified as group 3M members.

**Table 2:** Description of ribosomal gene arrays of the fibroblast cell lines studied.

Cells	Donor age	rDNA CN ± SE		*M* ± SE	
		Leukocyte	Fibroblast (5 p)	Leukocyte	Fibroblast (5 p)
HSF–45^∗^	35	430 ± 50	450 ± 40	1.1 ± 0.2	1.0 ± 0.1
HSF–53	21	390 ± 40	370 ± 20	1.0 ± 0.1	1.2 ± 0.2
HSF–57	52	570 ± 30	590 ± 40	2.1 ± 0.2	1.7 ± 0.2
HSF–61	52	480 ± 40	470 ± 60	1.2 ± 0.1	1.1 ± 0.2
HSF–66	35	640 ± 40	670 ± 70	2.2 ± 0.2	1.9 ± 0.2

*^∗^The digits in each line denotation indicated the order number of the final passage, after which the cells stopped their division. rDNA CN, rDNA copy number; SE, standard error.*

**FIGURE 4:**
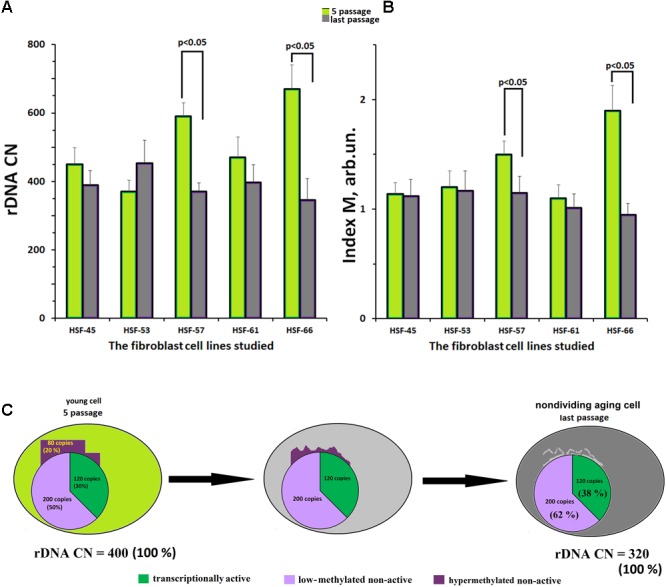
Replicative senescence of cultivated skin fibroblasts derived from five donors with known parameters of the ribosomal gene arrays. **(A)** rDNA content for the fifth and last passages of each of the five cell lines. **(B)** The methylation index *M* for the fifth and last passages of each of the five cell lines. Each value of the index *M* and rDNA CN represent the average of three independent experiments. **(C)** The scheme describing the loss of hypermethylated DNA copies with aging. We considered, as an example, a hypothetical genome containing 400 copies of rDNA.

**Figure 4A** indicates the values of rDNA CN for the fibroblast lines at the fifth and the final culture passages. In three of five cell lines, no change of rDNA CN during replicative senescence was observed. The other two lines (HSF–57 and HSF–66) showed a decrease in the rDNA CN with aging.

**Figure 4B** presents the values of methylation index *M* for the fibroblast lines at the fifth and at the final passages. In three fibroblast lines with initially low-methylated rDNA, no change of the methylation level was observed. However, in the other two lines with initially high content of hypermethylated copies (HSF–57 and HSF–66), a decrease in methylation was registered. At the final passage, no hypermethylated copies were detected in the rDNA of these lines.

Summarizing the results presented in **Figure 4**, one may conclude that the fibroblast genome almost completely loses its hypermethylated rDNA copies during the replicative senescence (**Figure 4C**).

## Discussion

The mean lifetime was 72 years in Russia as of 2016–2017. The principle result of this study: in a fraction of Russian residents, who have survived until the age of mean lifetime and older (E-group, 72–91 years old), the range of the rDNA CN (**Figures 2B,C**) in the genome was considerably narrower than in the group of younger subjects. Therefore, in NE-group the range was 200 to 711 rDNA CN, whereas in E-group, rDNA CN varied within a range of 272 to 541 copies. The range narrows to 311–482 rDNA CN for the people aged 83 to 91 years. Meanwhile, the mean values of rDNA CN did not differ (**Table 1**, *p* > 0.05). In the NE-group, 10% of the subjects carried less than 272 copies of rDNA, and 15% of the subjects carried more than 541 copies of rDNA.

### Genomes Harboring Small Numbers of rDNA Copies

Individuals with low rDNA CN seem to fail to supply the cell with the sufficient amount of ribosomes when a metabolic response to various exo- and endogenous stress factors including those associated with aging is required. One can speculate that the lowest total rDNA CN is correlated with a low number of active copies (fraction 1, **Figure 1**). *In vitro* experiments on the cell cultures showed that the efficiency of cell’s response to the action of a factor inducing oxidative stress and DNA damage directly depended on the number of active rDNA copies in the cell’s genome. The less rDNA copies in the genome, the more cells die after a toxic impact (Veiko et al., 2005). Also, we previously found small quantities of active rDNA copies in blood lymphocytes from patients with rheumatic arthritis (Lyapunova et al., 2013). This disease is characterized by reduced lifetime (Wong et al., 2001). Recently, it has been shown that a decrease in the rDNA CN in the cancer genomes correlates with an increase in the sensitivity of the cells to damaging factors (Xu et al., 2017; Udugama et al., 2018). These authors also believe that a low rDNA content is the cause of chromatin instability in the cells.

Thus, it can be expected that too low rDNA content in the human genome (<270 copies) is associated with a shorter lifetime. If this is true, then the low total rDNA CN in the genome can be considered as a marker of low life expectancy. This requires further research.

### Genomes Harboring High Numbers of rDNA Copies

Two hypotheses can be put forward to explain the existence of an upper limit (541 copies) for rDNA CN in E-group: (1) individuals with high rDNA CN in their genomes do not survive till the age of the population’s mean lifetime and/or (2) the human genome loses a part of rDNA copies with aging.

#### Individuals With High rDNA CN May Not Survive Until the Age of 72 Years

We can make some speculative assumptions. First, the embryonic lethality presumably creates a selective mechanism, which allows survival of aberrant karyotypes only if they have high rDNA abundance (Lyapunova et al., 2017). Higher numbers of rDNA copies in the genomes of schizophrenia patients can be also explained by this reason (Chestkov et al., 2018). In other words, detrimental in terms of survival and expected lifetime is not the high rDNA genomic dosage itself, but the factors of unfavorable genetic background, which require an elevated level of rDNA.

Second, a large total amount of rDNA in some cases correlates with a large number of active copies of rDNA (Veiko et al., 1996, 2003). A large number of active copies of rDNA can be toxic to the cells. Intriguing data were obtained during an investigation of Hutchinson–Gilford progeria syndrome (HGPS) caused by a mutation in the gene for lamin A protein (Eriksson et al., 2003). This mutation was accompanied by the derepression of rDNA transcription in the cells of HGPS patients (Goldman et al., 2004; Shumaker et al., 2006). The derepression of rDNA transcription is manifested in augmentation of the amount of mature forms of rRNA. The exaggerated production of rRNA in turn leads to a considerable increase in the production of ribosomal proteins. The total level of protein synthesis is also heightened significantly in the cells of HGPS patients (Buchwalter and Hetzer, 2017). The ribosome biogenesis and the protein translation are very energy-consuming processes, which require a great amount of ATP (by some estimates, up to 80% of the total cellular ATP) (MacInnes, 2016). Some authors suppose that the overproduction of proteins in the cells of HGPS patients entails premature aging due to the deficit of energy exhausted by this process. In the cells of HGPS patients, very low ATP levels and elevated metabolism rates are detected (Abdenur et al., 1997; Merideth et al., 2008). It is obvious that the large number of rDNA copies, in some cases can evoke the same response: a lack of energy because of the upregulated ribosome biogenesis and protein synthesis. This assumption is also proved by the fact that the number of rDNA copies in the genome negatively correlates with the abundancy of mitochondrial DNA (Gibbons et al., 2014).

In this context, a study reported by Tiku et al. (2016) is of interest. The authors showed that an increase of lifetime reached by means of various techniques, including calorie restriction, resulted in a decreased expression of both rRNA and ribosomal proteins. Deceleration of ribosome biogenesis and protein biosynthesis is viewed as a means to increase life expectancy. Accordingly, those individuals may have better longevity, whose genomes contain medium numbers of rDNA copies, which provide for the optimum level of ribosome biogenesis.

#### The Human Genome May Lose a Part of rDNA Copies With Aging

A number of authors (Johnson and Strehler; 1972; Gaubatz and Cutler, 1978; Zafiropoulos et al., 2005) put a hypothesis that links aging to loss of rDNA copies in the course of life forward. True, other authors did not report a decline in rDNA CN with age (Peterson et al., 1984; Halle et al., 1997; Machwe et al., 2000). Unfortunately, we cannot test this hypothesis directly, because we do not have a series of DNA samples of the same donors in different years within their lifespan. Therefore, we examined replicative senescence, in order to evaluate in principle on this model, if the loss of a fraction of rDNA copies exists during senescence. We assumed, that if some copies of rDNA is lost with aging/senescence, they can be the hypermethylated copies only (fraction 3, **Figure 1B**), which are situated at the periphery of the nucleolus (or outside the nucleolus), mutated largely, and cannot be re-activated at all. The hypermethylated rDNA copies can be found in some genomes, but not in all. They are a specific kind of ballast for the genome. From NE-group, five donors were selected, who contained different total rDNA CN and different number of the hypermethylated rDNA copies in leukocyte DNAs. Cell lines were obtained from skin biopsy samples of these donors. It is notable that at passage 5 of the cultivated skin fibroblasts both total number of rDNA copies and methylation index *M* were not changed in comparison with leukocytes. We revealed, that with replicative senescence, a loss of rDNA is only observed in the two cell lines with a high content of the hypermethylated rDNA copies (lines HSF–57 and HSF–66, **Figures 4A,B**). The loss of a fraction of rDNA was followed by a decrease of methylation index *M*, which at the final passages did not differ from methylation index *M* for rDNA in the other three fibroblast line’s DNA samples.

Thus, it cannot be excluded that hypermethylated rDNA copies can be lost in the course of human aging (**Figure 4C**). The rDNA is difficult to replicate and the loss of the ballast repeats might facilitate a successful cell cycle (Xu et al., 2017). Probably, it is the loss of hypermethylated copies with aging, which can be the only realistic explanation for the different contents of methylated rDNA copies in NE- and E-groups. The loss of hypermethylated rDNA may explain the absence in the E-group individuals with a high (>541 copies) rDNA CN, **Figure 4C**.

Thus, it is not yet clear, is the dynamics of rDNA abundance in the genome just a marker of aging or one of the causes that directly influences, positively or negatively, the individual lifetime? For the final conclusion on the causes of the narrowing range of rDNA CN with human aging, further studies are necessary with the use of DNA samples regularly collected from the same individuals during their lifetimes for a long period of time.

## Author Contributions

SVK, NV, VG, and SIK designed the study. EM, EE, and NV performed the experiments. EE and LP performed the statistical analysis. VG, NL, and SIK provided the human blood samples. EM provided cell cultures. SVK, LP, and NV wrote the initial draft. LP translated the manuscript to English. All the authors participated in critical revision and approved the final manuscript before submission.

## Conflict of Interest Statement

The authors declare that the research was conducted in the absence of any commercial or financial relationships that could be construed as a potential conflict of interest.

## Footnotes

1 http://www.analystsoft.com/
